# A review on function and side effects of systemic corticosteroids used in high-grade COVID-19 to prevent cytokine storms

**DOI:** 10.17179/excli2020-3196

**Published:** 2021-02-15

**Authors:** Mohammad Amin Langarizadeh, Marziye Ranjbar Tavakoli, Ardavan Abiri, Ali Ghasempour, Masoud Rezaei, Alieh Ameri

**Affiliations:** 1 Student Research Committee, Kerman University of Medical Sciences, Kerman, Iran; 2 Department of Medicinal Chemistry, Faculty of Pharmacy, Kerman University of Medical Sciences, Kerman, Iran; 3 Faculty of Medicine, Kerman University of Medical Sciences, Kerman, Iran

**Keywords:** COVID-19, cytokine storm, corticosteroid therapy, SARS-CoV-2, adverse effects

## Abstract

In December 2019, a cluster of pneumonia caused by a novel coronavirus (2019-nCoV), officially known as severe acute respiratory syndrome coronavirus 2 (SARS-CoV-2), emerged in Wuhan, Hubei province, China. Cytokine storm is an uncontrolled systemic inflammatory response resulting from the release of large amounts of pro-inflammatory cytokines and chemokines that occurs at phase 3 of viral infection. Such emergence led to the development of many clinical trials to discover efficient drugs and therapeutic protocols to fight with this single-stranded RNA virus. Corticosteroids suppress inflammation of the lungs during the cytokine storm, weaken immune responses, and inhibit the elimination of pathogen. For this reason, in COVID-19 corticosteroid therapy, systemic inhibition of inflammation is observed with a wide range of side effects. The present review discusses the effectiveness of the corticosteroid application in COVID-19 infection and the related side effects of these agents. In summary, a number of corticosteroids, including and especially methylprednisolone and dexamethasone, have demonstrated remarkable efficacy, particularly for COVID-19 patients who underwent mechanical ventilation.

## Introduction

Coronaviruses (CoVs) are a diverse group of enveloped viruses of the order *Nidovirales* and the *Coronaviridea* family. These positive-sense viruses with single-stranded RNA can manifest themselves by a disturbance in the gastrointestinal tract, hepatic activity, neurological network, and in particular, the respiratory system (Mao et al., 2020[76]; Wong et al., 2020[139]). In addition to humans, the virus has the potential to infect animals, that is the main reason for transferring of coronaviruses, including COVID-19 from zoonotic sources (Loo et al., 2020[73]). CoVs are divided into four main genera including alpha, beta, gamma, and delta. SARS-CoV and MERS-CoV are two new β-CoVs that have caused a series of lethal respiratory illnesses in the last two decades (Zumla et al., 2016[154]; Xu et al., 2020[142]). With the sudden outbreak of the novel coronavirus in the late December 2019 in the Wuhan region and its rapid globalization, human society has failed to achieve a consensus cure for the disease, and unfortunately, the casualties caused by this pandemic are increasing daily. Due to the lack of sufficient knowledge in pharmacotherapy to treat the disease, different treatment processes were implemented in terms of drug type, method of use, and duration of treatment around the world (Rothan and Byrareddy, 2020[108]; Zhou et al., 2020[151]). COVID-19 appears to be more contagious than SARS and MERS, but fortunately, it is less lethal, killing only 2.2 % of sufferers (Rothan and Byrareddy, 2020[108]; Zhou et al., 2020[153]). Most patients have mild symptoms, but according to the Chinese government, about 13.2-21.3 % of patients experience critical conditions like septic shock, acute respiratory distress syndrome (ARDS), progressive pulmonary insufficiency, pulmonary edema, severe pneumonia, or death (Sohrabi et al., 2020[124]; Xu et al., 2020[142]; Zhou et al., 2020[151][153]). Hydroxychloroquine and chloroquine were transiently approved by the US Food and Drug Administration (FDA) for emergency usage in critically-ill patients, which was later revoked. On October 22, 2020, FDA confirmed Veklury® (remdesivir) for the treatment of COVID-19 patients (FDA, 2020[41]). A bit later, in November 2020, this organization approved the combination therapy of remdesivir with baricitinib for the treatment of suspected or confirmed cases of COVID-19 (patients of two years of age or older) (FDA, 2020[39]). To date, dexamethasone is the only certified corticosteroid medication that is recommended for hospitalized patients requiring supplemental oxygen (including those who need high flow oxygen or mechanical ventilation) by a survey conducted in the University of Oxford (FDA, 2020[40]). Certain antiviral medicines or vaccines are yet to be approved for COVID-19 infection, but now unofficially, more than 30 different drugs are being used with experimental or proven effects, including herbal compounds, synthetic drugs, and traditional Chinese medicine (Rothan and Byrareddy, 2020[108]). Most of the drugs used by medical staff in different regions are antiviral drugs that inhibit the vital proteins of coronavirus, including papain-like protease, RNA dependent RNA polymerase, 3C-like proteinase, and spike protein (or its receptor, ACE2), which prevent the progression of the disease (Rismanbaf, 2020[103]). The fifth trial version of the Diagnostic-Therapeutic scheme for controlling the pneumonia-like symptoms of COVID-19 infection was drafted by the China National Health Commission on February 7, 2020, and provided a systematic treatment strategy for intense cases of sufferers. Corticosteroids were used as adjunctive therapy in this scheme. More precisely, methylprednisolone, 1-2 mg/kg daily for 3-5 days, was recommended (Zhou et al., 2020[153]).

## Methodology

This work was done according to focusing on the role and manner of effectiveness, clinical applications, and side effects of corticosteroids in various forms to reduce mortality of COVID-19. The required searches are done in Google scholar and PubMed search engine. To search for the clinical application of each drug in COVID-19, the combined keywords [drug name] + [COVID-19 or SARS-CoV-2] have been used. Also, to search for the side effects of each drug, combined keywords including [drug name] + [side effects] have been used. PubMed searches were limited to advanced search in titles. Other searches have been done based on the general focus of the content of each section or on an *ad hoc* basis. The coherence of the text from a scientific and literary point of view was then re-evaluated and then revised accordingly.

## Mechanism of Immune Cell Response

### Innate immune response

The entrance of the virus to the body is mediated by ACE-2 receptors on the surface of the epithelial cells of the respiratory tract through spike (S) proteins. The virus will then start to replicate, followed by spreading through the lower respiratory tract that eventually triggers a robust immune response. The infected cells produce inflammatory cytokines in the lungs that attract the nearby macrophages (Roh and Sohn, 2018[106]; Li et al., 2020[68]). The alveolar macrophages are M1 and M2 types with pro-inflammatory activity and regulatory functions, serving as innate immunity players in the lung (Li et al., 2020[68]). Coronavirus directly infects macrophages and T cells. Innate immune cells have pattern recognition receptors (PRRs) that can recognize the virus invasion by their hallmarks called pathogen-associated molecular patterns (PAMPs). Interaction between Toll-like receptors (TLRs, one of the most important PRRs) in the lungs and nucleic acid of the virus found to activate immune response through the production of antibodies by B cells and release of interferons (Dhama et al., 2020[29]; Jamwal et al., 2020[57]). The performed investigations on patients who survived from coronavirus have revealed an extreme expression of IFN-ɑ, IFN-γ, CXCL10, and CCL2. Furthermore, elicitation of the immune response has been also observed via gene expression analysis. Some studies in Wuhan illustrated an increased level of neutrophils and serum IL-6 but decreased levels of lymphocytes. In many patients, the elevated plasma level of some innate cytokines, such as monocyte chemo-attractant protein-1 (MCP-1), macrophage inflammatory protein 1alpha (MIP-1A), and TNF-ɑ, have been observed. IFN response is the most effective innate immune response to defense, prevents the spreading of the virus and also induce the development of adaptive immune response, and promotes macrophage, natural killer (NK), T and B cells' functions (Dhama et al., 2020[29]).

### Adaptive immune response

The adaptive immune system consists of humoral and cellular immunity. This response is confirmed by CD8^+^, CD4^+^ T cells, and B cells that are adapted to specific pathogens. This level of immune response happens (IFN-ɑ or anti-sera) during the non-severe stages to prevent disease advancement to severe stages. Genetic differences in people make the individual variation in the adaptive immune response to the virus, including T cells, which eliminate the infected cells and pathogens, and B cells, which produce antibodies for specific pathogen and will be then differentiated to generate immunoglobulin (Ig). All immunoglobulins (IgM, IgA, and IgG) have been seen in the serum of SARS-CoV infected patients. Based on the previous surveillances, IgM (which is the firstly secreted in response to acute inflammation) and IgG antibodies can remain in the plasma for a while and play a protective role. Recent studies firmly showed the critical role of T helper 1 (Th1) type response to limit SARS-CoV and MERS-CoV infections (Dhama et al., 2020[29]; Ye et al., 2020[147]).

In the following, the mechanism of action, indications, and side effects of systemic corticosteroids (i.e., those corticosteroids that are given orally or by injection (not topically) and are distributed throughout the body) used for COVID-19 patients will be explored. The side effects of inhaled corticosteroids are listed separately below.

## Mechanism of Action

Corticosteroids can prevent lung injury caused by severe community-acquired pneumonia (sCAP) due to their immunomodulatory and anti-inflammatory properties (Stockman et al., 2006[127]; Zhou et al., 2020[153]). These agents restrict the inflammation (which is the leading cause of severe lung damage and delayed recovery) by reducing the excessive production of inflammatory cytokines, chemokines, and activated lymphocytes (Ni et al., 2019[89]). Cytokine storm is one of the leading causes of disease exacerbation and progression to ARDS, which is the main cause of death in COVID-19 patients. Prior to the coronavirus pandemic, there was evidence of cytokine storms in viral infections. H5N1, dengue virus, Ebola virus, SARS-CoV, EBV, DHAV-1, Zika virus, and human herpesvirus are among the viruses that cause hemophagocytosis, cell damage, and organ dysfunction by causing hypercytokinemia (Imashuku, 2002[52]; Huang et al., 2005[50]; Us, 2008[136]; Wu et al., 2008[140]; Rothman, 2011[109]; Sordillo and Helson, 2015[125]; Li et al., 2018[69]; Xie et al., 2018[141]; Maucourant et al., 2019[79]). The best time to prescribe corticosteroids in the treatment of COVID-19 patients is when the patient's condition is deteriorating, i.e., progressing to ARDS. Some patients usually have a sudden worsening 1-2 weeks after the onset. Increased resting respiratory rate, drop in oxygen saturation level when the person is breathing the room air, and multi-lobular progression on imaging within 48 h are some good indicators of the right time to take glucocorticoids (Zhou et al., 2020[152]). Meanwhile, the rapid and short-term initiation of anti-inflammatory therapy during this short period of time can most likely lead to an acceptable therapeutic response (Ahn et al., 2020[2]; Zhang et al., 2020[149]). According to the clinical signs and findings, the disease is divided into three stages with different severity. The first stage is related to the early exposure of the body to the virus, which is an emerging infection with nonspecific symptoms (malaise, fever, and dry cough), all of which are related to the incubation of the virus and the development of the disease. Respiratory polymerase chain reaction, serum testing for virus-related IgG and IgM, chest imaging, complete blood count, and liver function tests are among the diagnostic methods at this stage. Antiretroviral pharmacotherapies, such as remdesivir or favipiravir, are the best treatment options at this stage which is called early infection or viral response phase (Siddiqi and Mehra, 2020[121]). In the second stage (pulmonary phase), virus multiplication and localization of the disease and inflammation in a number of areas of the body, such as the lungs, may occur, which can lead to hypoxia. Bilateral infiltrates, ground-glass opacities in radiography, increasing lymphopenia, and transaminitis may help to diagnose this stage. At this point, corticosteroids should be avoided, and antiretroviral therapy and supportive measures should be considered unless the hypoxia ensues. If hypoxia occurs, mechanical ventilation and the use of anti-inflammatory drugs such as a low to moderate dose of corticosteroids may be effective for the patient (Shang et al., 2020[119]; Siddiqi and Mehra, 2020[121]). The third stage is the most severe stage of the disease, which can lead to ARDS and death. Systemic inflammation, increased inflammatory markers, decreased T lymphocyte count, and lymphocytopenia are important symptoms of this stage that eventually lead to multiple organ failure. Anti-inflammatory and immunomodulatory agents such as glucocorticoids, immunosuppressants, inflammatory cytokines antagonists such as tocilizumab (IL-6 receptor inhibitor) or anakinra (IL-1 receptor antagonist), and intravenous immune globulin (IVIG) can be helpful in this stage while antiviral drugs may not be effective (Siddiqi and Mehra, 2020[121]; Zhang et al., 2020[149]). Timely use of glucocorticoids can improve fever and provide better oxygen delivery, but some studies have suggested that the use of these agents is incorrect due to weakened immune response and reduced virus clearance (Zhang et al., 2020[149]). Combination therapy with ribavirin and corticosteroids has been experimentally effective and, in fact, has a scientific basis. In addition to being effective in treating respiratory syncytial virus infection, influenza virus A and B infections, measles, parainfluenza, and Lassa fever, ribavirin has been found to inhibit RNA-dependent RNA polymerase (Oba, 2003[94]; Elfiky, 2020[35]). In acute viral respiratory infections, rapid-acting cytokines and inflammatory markers such as IL-1β, IL1RA, IL-2, IL-4, IL-6, IL-7, IL8, IL9, IL-10, TNF-α, CRP, ferritin, D-dimer, IFN-γ, IP-10, MCP-1, basic FGF2, GCSF, GMCSF, MIP1α, MIP1β, PDGFB, and VEGFA mediate the lung damage. IL-2, IL-7, IL-10, G-CSF, TNFα, IP-10, MCP-1, and MIP-1A were higher in critically-ill patients such as those in intensive care unit (ICU) (Fu et al., 2020[43]; Huang et al., 2020[49]; Nile et al., 2020[91]; Siddiqi and Mehra, 2020[121]; Zhang et al., 2020[149]). Glucocorticoids with a genomic mechanism inhibit the synthesis of pro-inflammatory cytokines such as IL-1, IL-2, IL-6, IL-8, TNF, IFN-gamma, COX-2, VEGF, and prostaglandins (Dinarello, 2010[31]). Prednisolone, for example, inhibits the production of TNF, IFN-γ, IL-1β, IL-6, IL-17, IL-10, and TGF-β. Dexamethasone also significantly reduces the level of IL-6, which is very problematic in critically-ill patients. Corticosteroids also suppress inflammation by non-genomic mechanisms such as i) binding to the membrane-associated glucocorticoid receptors of T cells resulting in perturbation of receptor signaling and immune response and ii) interacting with the exchange of calcium-sodium across the cell membrane, resulting in a quick downturn in inflammation (Levine et al., 1993[67]; Negera et al., 2018[86]; Yasir et al., 2020[145]).

Therefore, to prevent the progression of lung disease, corticosteroid therapy is used to suppress the cytokine storm. Previous surveys show that lung opacities in X-ray began to fade and oxygen delivery started to improve after corticosteroid therapy (Oba, 2003[94]). Acute lung damage and acute respiratory distress syndrome are partly due to the immune response and inappropriate inflammatory mediators in the host. Corticosteroids not only suppress the inflammation of the lungs during the cytokine storm, but also, they weaken immune responses and inhibit the elimination of pathogens. For this reason, in COVID-19 patients, corticosteroid therapy (similar to influenza) systemic inhibition of inflammation is associated with a wide range of side effects. Thus, in patients experiencing life-threatening jeopardies in the late stages of COVID-19, corticosteroid therapy may be a risk-benefit option, and it might not be a reliable choice for everyone (Russell et al., 2020[110]). Another reason why corticosteroids are controversial is that, according to research, patients taking corticosteroids may develop a variety of secondary infections, including bacterial respiratory infections, due to suppression of the immune system. Risks such as shock, cardiovascular events, fluid retention, premature atherosclerotic disease, and arrhythmia are also more likely to occur in patients treated with corticosteroids (Ni et al., 2019[89]). Corticosteroids are not recommended in the early stages of the disease owing to delayed recovery, especially when treatment with antiviral drugs has not yet been performed. This recommendation is based on a study that vindicated early consumption of weak corticosteroids such as hydrocortisone, even at low doses, in more than 80 % of cases, has not been able to stop the progression of the disease (Lee et al., 2004[64]). Thus, corticosteroid therapy can be effective only in the high stages of the disease and in certain conditions in combination with antiviral drugs, in which monitoring the patients' condition is essential to prevent adverse events.

Among all, hydrocortisone and prednisone have the highest protein binding (Lester et al., 1998[66]; Czock et al., 2005[21]). Cortisol with 8 to 12 hours has the shortest, and dexamethasone and betamethasone with 36 to 54 hours have the longest biological half-life (Melby, 1977[80]). Some corticosteroids have a number of mineralocorticoid properties, the potency of which is directly related to some side effects such as water and sodium retention, edema, hypocalcemia, fluid-electrolyte disturbance, elevated calcium excretion, weight gain, and hypertension (Melby, 1977[80]; Lester et al., 1998[66]; Czock et al., 2005[21]).

## General Side Effects of Corticosteroids

**Figure 1:**
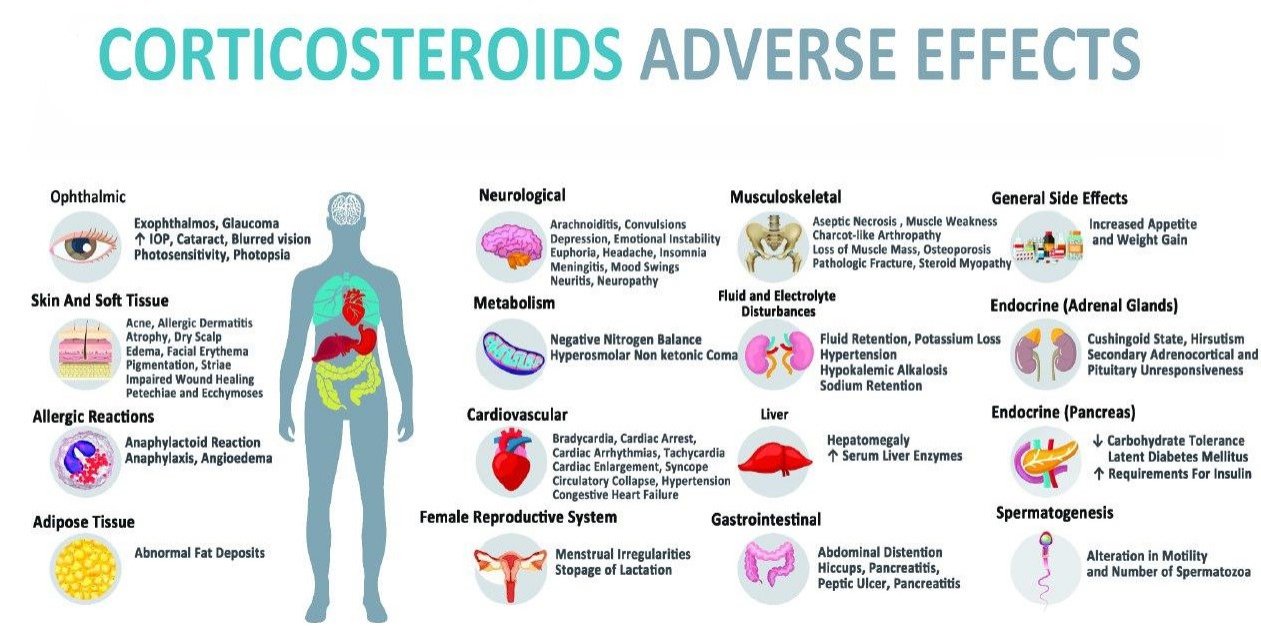
General side effects of corticosteroids with location in the body

In addition to the specific side effects of each corticosteroid, this category also has a number of general adverse effects that represent the typical features of this whole class of medications. Common side effects of corticosteroids vary, and a wide spectrum of complications such as fluid retention, changes in glucose tolerance, behavioral and mood alteration, weight gain, high blood pressure, and increased appetite was recorded. Basically, these general side effects are divided into twelve categories; cardiovascular system, dermatological complications, endocrine glands, fluids, and electrolytes balance, gastrointestinal tract, renal system, metabolism, musculoskeletal system, nervous system, ophthalmic complications, reproductive system, and allergic reactions (FDA, 1955[42]; Melby, 1977[80]; Berthelot et al., 2013[10]; Yu et al., 2018[148]). Complications and symptoms associated with each of these reactions are depicted in Figure 1(Fig. 1).

## Specific Side Effects of Corticosteroids

### Methylprednisolone

Methylprednisolone has a prominent position among corticosteroids and is used to treat many dangerous illnesses, including numerous autoimmune diseases. Methylprednisolone reduces mortality by up to 71 % in patients with COVID-19 and reduces the need for a ventilator (Salton et al., 2020[112]; Papamanoli et al., 2021[95]). Also, in patients who were expected to require mechanical ventilation, the number of days without ventilation was increased and extubation was more likely (Nelson et al., 2020[87]). In comparison, the efficacy of methylprednisolone and dexamethasone has been reported to be the same in moderate to severe cases (Fatima et al., 2020[38]), however, methylprednisolone is sometimes better than dexamethasone in preventing mortality due to its better pharmacodynamics and pharmacokinetics (Liu et al., 2020[72]). On the other hand, there have been reports that dexamethasone is more effective in reducing C-reactive protein (CRP) and improving P/F ratio (pO_2 _divided by the fraction of inspired oxygen (FIO_2_)) (Rana et al., 2020[100]). Early use of low-dose methylprednisolone not only prevents the progression of the disease in critically-ill patients, but also reduces the incidence of ARDS and death (Yang et al., 2020[144]). Timely and short-term use of methylprednisolone is crucial, as a cohort study propounded that a treatment period of more than 7 days with methylprednisolone can increase the risk of death in the patients (Ji et al., 2020[60]). In a double-blind study of 647 adults with COVID-19 in Brazil, the death rate of people over 60 in the first 28 days of intravenous methylprednisolone was lower than that of the placebo group (Jeronimo et al., 2020[59]). A variety of combination therapies also seem to be effective. Due to the important role of interleukin-1 (IL-1) in coronavirus inflammation, concomitant use of the IL-1 antagonist Anakinra and methylprednisolone reduced mortality by 21.7 % compared to the control group (Bozzi et al., 2021[12]). Also, the combined use of favipiravir and methylprednisolone early on 11 cases of COVID-19, all in critical condition and undergoing oxygen therapy, resulted in a recovery of 10 patients (~91 %) (Murohashi et al., 2020[84]). Tocilizumab, an IL-6-blocking monoclonal antibody, along with methylprednisolone, has been shown to be effective in patients with severe conditions (Sanz Herrero et al., 2021[113]). High dose methylprednisolone (a single bolus of 250 mg, followed by 80 mg on days 2-5) has been used successfully to rescue critically-ill patients who were hospitalized in the ICU who have not responded to azithromycin, hydroxychloroquine, and even two doses of tocilizumab (Conticini et al., 2020[20]). MATH+ medication regimen has been suggested as one of the effective protocols in the pulmonary phase of the disease, which includes methylprednisolone, ("M"), high-dose vitamin C infusion ("A"), thiamine ("T"), heparin ("H"), ivermectin, and supplemental components ("+") such as melatonin, vitamin D, zinc, and magnesium (Turkia, 2020[135]). In clinical trials, there are a series of certifications for creating rhythmic complications in its injectable dosage form, including sinus bradycardia, atrial fibrillation, atrial flutter, and ventricular tachycardia. Incidents such as hypertension, hyperglycemia, and fluid-electrolyte disturbances may also occur during low-dose methylprednisolone therapy (Darling et al., 2013[24]). Unlike cardiovascular events such as severe hypertension, myocardial infarction, acute heart failure, angina pectoris, ischemic stroke, and pulmonary embolism, which occur more frequently in lower doses (2.0-5.0 g), the incidence of unwanted hepatic symptoms such as acute liver failure is more probable at higher doses (> 5.0 g). Intravenous methylprednisolone cumulative dose should not exceed 8 grams due to severe side effects including hepatic toxicity and elevated liver enzymes (Walasik-Szemplińska et al., 2019[138]). It may be necessary to take antibiotics and antifungals after treatment with methylprednisolone, as several cases have been reported that by restraining the immune system, a variety of bacterial and fungal infections of the urinary tract, vagina, and mouth may occur. After prescribing methylprednisolone, there is a possibility of early (12 hours to 1 month) or late (even up to 4 years) epilepsy. Due to the occurrence of glucosuria and hyperglycemia in many patients treated with methylprednisolone, administration in diabetic and pre-diabetic patients should be followed with caution. Gastrointestinal side effects, such as nausea, duodenitis, and persistent pain or heartburn associated with stomach acid, are possible. Among the psychological side effects, depression or euphoria are most likely to manifest (Lyons et al., 1988[75]). In very rare cases of methylprednisolone administration, severe side effects occur and intense adverse events are usually scarce. In a group study related to measuring the effectiveness of intravenous methylprednisolone in patients with COVID-19 in Iran, out of 68 patients who entered the clinical trial randomly and in a controlled manner, only 2 developed severe side effects (Edalatifard et al., 2020[34]). 

### Prednisolone 

Prednisolone has fewer side effects than other corticosteroids in a short duration of therapy with high concentrations or acute overdoses. In fact, short-term treatment with prednisolone is very unlikely to have significant side effects. *In silico* studies based on molecular docking and dynamics revealed that dextromethorphan combined with prednisolone or dexamethasone could have a synergistic effect on inhibition of the virus main protease (M^pro^), but its efficacy was not studied *in vitro* (Sarkar and Sen, 2020[114]). Gastrointestinal complications, including nausea, vomiting, abdominal distension, and elevated appetite are common. Increased motor activity, insomnia, and agitation may also occur. Chronic prednisolone can suppress the pituitary-hypothalamic-adrenal pathway. In this case, with discontinuation of the drug, there is a possibility of acute adrenal insufficiency, which includes symptoms such as shock, anorexia, headache, fever, joint pain, hypotension, nausea, and vomiting. On the other hand, by changing the distribution of fat in the body, fat masses in the peripheral areas become reduced and instead accumulate in the central regions of the body, which causes a condition called buffalo hump appearance. Long-term use of prednisolone also engenders endocrine disorders such as Cushing's syndrome, amenorrhea, and menstrual disorders. The patient's history and the risk factors for diabetes must be considered because taking prednisolone reduces glucose tolerance and causes hyperglycemia, which can lead to diabetes mellitus. Based on what we expect about the structure-activity relationship (SAR) of corticosteroids, prednisolone has some mineralocorticoid activity, which is the reason for complications such as hypocalcemia, edema, fluid-electrolyte disturbance, fluid retention, elevated calcium excretion, and weight gain (Blake, 1990[11]; Robinson et al., 2016[104]). Other disorders observed in patients using prednisolone are delineated in Table 1(Tab. 1).

### Dexamethasone

Dexamethasone was first prescribed to patients in COVID-19 by British physicians, and after conducting the necessary research, RECOVERY (Randomised Evaluation of COVid-19 thERapY) trial was set. In this trial, low-dose dexamethasone was injected in a dose of 6 mg daily for 10 days to minimize the incidence of side effects along with proper effectiveness (Cain and Cidlowski, 2020[16]). According to this trial, taking dexamethasone will be effective only for people who receive respiratory support. The dexamethasone-related mortality reduction was about 35 percent for people who were being ventilated and about 20 percent for people receiving oxygen therapy (Lester et al., 2020[65], RECOVERY Collaborative Group et al., 2020[101]). A very important point about taking dexamethasone and other corticosteroids is that people may seek self-medication for fear of illness with insufficient information, which in turn can lead to a number of damages related to the side effects of these drugs. Therefore, at this time, it is necessary for health professionals to be vigilant to minimize the potential harm and be able to predict the side effects caused by these drugs (Alessi et al., 2020[3]). A prominent therapeutic effect of dexamethasone in COVID-19 is reduced vascular permeability and prevention of myocardial edema (Rafiee et al., 2020[99]). Based on another mechanism, following viral infection, an increase in pro-resolving lipid mediators such as protectins, resolvins, maresins, and lipoxins is probable and dexamethasone may block the activity of these mediators (Andreakos et al., 2020[4]). Also, according to a hypothesis based on computational studies, dexamethasone prevents virus entrance by occupying the SARS-CoV-2 spike pseudotyped virus binding site in the ACE2 (Zhang et al., 2021[150]). In a randomized controlled trial of 2104 hospitalized patients, dexamethasone was found to reduce mortality, but this effect only affects patients receiving oxygen and mechanical ventilation and does not include people without respiratory support (RECOVERY Collaborative Group et al., 2020[101]). A similar study in 299 people in Brazil found that the dexamethasone group had more ventilator-free and ICU-free days than the placebo group. There were also fewer adverse events and secondary infections in these individuals (Tomazini et al., 2020[132]). Combination therapies with dexamethasone can be effective. Due to the weakening of immune mechanisms and the possibility of reduced virus clearance after dexamethasone, its combined use with intravenous immunoglobulin and beta interferon may be a viable option (Abdolahi et al., 2020[1]). Concomitant use of dexamethasone with long-acting beta-2 adrenergic agonists such as salmeterol and formoterol relieves respiratory symptoms and together synergistically improves the anti-inflammatory and anticoagulant effects (Hajjo et al., 2020[46]). Also, in a controlled study with a treatment regimen consisting of inhaled corticosteroids along with remdesivir and dexamethasone, 5 out of 6 patients survived (Yatam Ganesh and Nachimuthu, 2020[146]). In a special case, a pregnant woman with COVID-19 was successfully treated with dexamethasone, remdesivir, convalescent plasma, and mechanical ventilation at 26 weeks of gestation (Jacobson et al., 2021[55]). Measurement of blood ferritin activity in patients can play an important role in determining the time window of dexamethasone administration, because the level of ferritin in patients who die is much higher than those who recover, and in fact can be a good criterion for assessing cytokine storm (Burugu et al., 2020[15]). The use of the leukosomal form of dexamethasone, which is a type of nanovesicle, exhibited good results *in vitro* and in this formulation dexamethasone had better therapeutic activity (Molinaro et al., 2020[82]). Dexamethasone is transported in the body through binding to serum albumin. The binding site for dexamethasone in albumin is the same with testosterone and NSAIDs, and therefore this competition should be considered (Shabalin et al., 2020[116]). Dexamethasone is well tolerated as a widely used corticosteroid in short-term or single-dose use, but with increasing the dose or duration of therapy, a number of side effects appear. Side effects such as glucose intolerance and hyperglycemia, increased risk of infection, especially fungal infections, delayed wound healing, adrenal suppression, joint avascular necrosis, gastrointestinal bleeding and perforation, restlessness, flushing, and to a lesser extent, nausea and vomiting are possible after taking dexamethasone (De Gans and Van Beek, 2002[25]; Thomas and Beevi, 2007[131]; Batistaki et al., 2017[8]). Decreased sleep quality, anxiety, insomnia, increased sweating, hirsutism, cutaneous purpura, and facial rounding are considered as minor side effects of dexamethasone (Bunim et al., 1958[14]; Dinan et al., 1997[30]). Advanced hypertension, edema, hyperglycemia, and glucosuria are significantly less likely to occur after dexamethasone consumption than other corticosteroids (Bunim et al., 1958[14]). Strongyloidiasis with symptoms of eosinophilia can be a hyperinfection in patients with COVID-19 following the use of dexamethasone, which can be prevented by taking ivermectin, which itself has an anti-coronavirus effect (Stauffer et al., 2020[126]). Dexamethasone-related information and its side effects are classified in Table 1(Tab. 1).

### Hydrocortisone

Among severe COVID-19 patients, treatment with a 7-day fixed-dose hydrocortisone or shock-dependent hydrocortisone dose resulted in a 93 % and 80 % improvement over 21 days, compared with no hydrocortisone treatment, respectively (Angus et al., 2020[5]; Prescott and Rice, 2020[97]). Low-dose hydrocortisone treatment in patients experiencing ARDS has mild positive results compared with the placebo group, and most of these trials have been discontinued early (Angus et al., 2020[5]; Dequin et al., 2020[28]). Hyperglycemia (glucose > 150 mg/dl) is more likely to occur after taking hydrocortisone, but it often does not lead to insulin administration. It is recommended to take antihypertensive drugs to control hypertension, which is likely to be followed by hydrocortisone. Secondary infections, muscle weakness, hypernatremia, and other general side effects associated with corticosteroids have not been significantly observed in patients who have taken hydrocortisone (Keh et al., 2016[61]). The structure and possible side effects of hydrocortisone are listed in Table 1(Tab. 1).

### Fludrocortisone

Fludrocortisone is a potent mineralocorticoid that retains sodium. Following this event, edema, weight gain, and hypertension are very likely. Therefore, the use of this drug in patients with a history of hypertension and other related diseases is not recommended. Also, the dose of the drug, the amount of salt consumed by the patient, and the serum level of the electrolytes should be monitored regularly. Allergic reactions and systemic infections have been reported in some studies. Antibiotic treatment is recommended to prevent infections along with fludrocortisone therapy (Glenmark Pharm Eur Ltd, 2017[44]). Cardiac complications include cardiac failure, systolic hypertension, and stroke. In some cases, evidence of depression has been found. Hyperkalemia is also a common side effect of long-term use of fludrocortisone, but it is not serious enough to halt the treatment protocol (Table 1(Tab. 1)) (Hussain et al., 1996[51]; Taplin et al., 2006[129]).

### Prednisone

Prednisone along with dexamethasone and methylprednisolone are among the drugs offered in the RECOVERY trial to reduce mortality in patients with hypoxemia. The dose of 0.5-1 mg prednisone per day for 3-4 weeks is considered for patients with COVID-19 (Bani-Sadr et al., 2020[6]; Mattos-Silva et al., 2020[78]). In a special case, a woman with severe Crohn's disease treated with prednisone and adalimumab was exposed to COVID-19 and received acceptable results as the two drugs continued to be administered (Vechi et al., 2020[137]). There are other similar results in another COVID-19 patient with autoimmune pancreatitis who was taking a high dose of prednisone, which may support the appropriate efficacy of this drug (Liaquat et al., 2020[70]). Prednisone has obvious bone complications, and a number of recommendations, namely lifestyle changes, should be applied to reduce the risk of osteoporosis (Shah and Gecys, 2006[117]). There is a high probability of unwanted incidences, but the treatment should be continued despite these adverse effects. The main problem with prednisone is fluid retention, which increases the volume or the frequency of urination. Gastrointestinal complications, including heartburn, diarrhea, and nausea, are also likely to be seen. Minor complications of prednisone in long-term consumption are myalgia, dermatologic events, insomnia, mood changes, bloating, hot flashes, joint swelling, depression or euphoria, elevated appetite, tremor, dizziness, swollen fingers, and changes in fat distribution (Table 1(Tab. 1)) (Lozada et al., 1984[74]; Ton et al., 2005[133]).

### Cortisone 

A number of gastrointestinal side effects have been reported with cortisone, including constipation, abdominal cramps, nausea, vomiting, elevated appetite, gastric ulcer, and diarrhea. Insomnia, mental stress, schizophrenia, and talkativeness are among the psychological side effects of cortisone. Other complications including hyperglycemia, moon-shaped face, mild acne, dizziness, glycosuria, headache, palpitations, weakness, edema, sodium retention, hypokalemia, hypertension, weight gain, osteoporosis, diabetes, infections, and atrophy of the adrenal gland are likely to appear (Table 1(Tab. 1)) (Schwartz et al., 1952[115]; Silltzbach, 1952[122]; Nagai, 1969[85]).

## Inhaled Corticosteroids (ICSs)

Today, corticosteroids, like many other drugs, come in a variety of dosage forms in the pharmaceutical market. Corticosteroids in the form of inhaled aerosols and powders are also suitable dosage forms for patients with lung diseases like COPD and asthma, which are marketed in three forms of metered-dose inhaler (MDI), dry powder inhaler (DPI) and nebulizers. High-volume spacer is also an auxiliary device that could be related to MDIs (Barnes and Pedersen, 1993[7]; Roland et al., 2004[107]; Irwin and Richardson, 2006[53]). As mentioned earlier, there are ambiguities about the types of inhaled corticosteroids and their use in COVID-19. Some sources claimed that respiratory patients with COVID-19 that take inhaled corticosteroids had more severe consequences than other patients (Nicolau and Bafadhel, 2020[90]). Conversely, according to a cohort study, inhaled corticosteroids in COPD patients were not associated with an enhanced risk of developing COVID-19 and even reduced the related risks in asthmatic patients (Choi et al., 2020[18]). This article supports the inhibitory effects of inhaled corticosteroids for several reasons. First of all, these compounds significantly reduce the risk of disease recurrence and progression to ARDS due to their regulatory role in inflammatory and immune responses (Halpin et al., 2020[47]; Nicolau and Bafadhel, 2020[90]). More importantly, inhaled corticosteroids have been shown to be associated with decreased gene expression of proteins ACE2 and TMPRSS2 in the epithelial cells of the oral mucosa and type 2 alveolar cells, thereby reducing the replication of coronaviruses, including SARS-CoV-2. ACE2 and TMPRSS2 are associated with the entry of the virus into the cell and are involved in the binding of the spike protein and the beginning of the viral infection cycle (Choi et al., 2020[18]; Nicolau and Bafadhel, 2020[90]). Complications of inhaled corticosteroids can be divided into local and systemic. Local complications versus systemic complications are minor problems but cannot be ignored (Roland et al., 2004[107]).

### Local side effects of inhaled corticosteroids

Local side effects are one of the most commonly reported problems with inhaled corticosteroids following the accumulation of particles in the upper respiratory tract (Barnes and Pedersen, 1993[7]; Hanania et al., 1995[48]). About 60 % of people treated with inhaled corticosteroids experience at least one local complication (Dubus et al., 2001[33]). In the following, we will examine the common local side effects of inhaled corticosteroids.

#### Oropharyngeal candidiasis

Oropharyngeal candidiasis is a dose-dependent complication that depends on the both total dose and the frequency of use. Taking it twice a day is less likely to cause candidiasis than taking the same dose four times a day (Barnes and Pedersen, 1993[7]; Hanania et al., 1995[48]; Toogood, 1998[134]). This complication is up to 70 % likely to occur and seems to be less likely in children (Barnes and Pedersen, 1993[7]; Hanania et al., 1995[48]; Roland et al. 2004[107]).

This complication is most often oral, and esophageal types are rarely seen. People with diabetes are much more prone to develop esophageal candidiasis (Irwin and Richardson 2006[53]). The main causes of this complication are actually a decrease in local immunity and an increase in salivary glucose levels, both of which can lead to oral thrush (Roland et al., 2004[107]). This complication is often self-limiting, but the cumulative dose or frequency of use can be reduced to minimize candidiasis. It has also been shown that rinsing the mouth and gargling after each use of the drug and using high-volume spacers can reduce the risk of thrush (Barnes and Pedersen, 1993[7]; Hanania et al., 1995[48]; Roland et al., 2004[107]). Amphotericin B and nystatin are also good pharmacological treatments. Oral thrush is usually well-tolerated and there is no need to stop the course of treatment (Barnes and Pedersen, 1993[7]; Hanania et al., 1995[48]). 

#### Dysphonia and hoarseness of the voice

Hoarseness is also a dose-dependent complication of inhaled corticosteroids, but unlike candidiasis, it is not frequency-dependent. It is very common and about one third to half of patients experience it (Barnes and Pedersen, 1993[7]). Not only does the use of high-volume spacers not reduce this complication but also it increases the likelihood of hoarseness (Barnes and Pedersen, 1993[7]; Irwin and Richardson, 2006[53]). Dysphonia is up to 50 % likely to occur and usually presents with candidiasis (Hanania et al., 1995[48]; Roland et al., 2004;[107] Irwin and Richardson, 2006[53]). The main reason is the movement disorder caused by the effect of steroids on the muscles that control the vocal cords (Hanania et al., 1995[48]). There are reports that dysphonia is less likely to occur with DPI than MDI (Barnes and Pedersen, 1993[7]; Roland et al., 2004[107]). This complication is usually reversible after the end of treatment period, but to reduce the likelihood of its occurrence, strategies such as reducing the dose, reducing vocal stress, and rinsing the mouth can be used (Barnes and Pedersen, 1993[7]; Hanania et al., 1995[48]; Roland et al., 2004[107]; Irwin and Richardson, 2006[53]). It seems that taking budesonide DPI twice a day, even in high doses, is less likely to cause dysphonia and candidiasis (Toogood, 1998[134]).

#### Cough, bronchospasm, and throat irritation

Cough is one of the most common complications of inhaled forms of corticosteroids, which is mostly due to the presence of excipients such as propellants and surfactants in the drug dosage form (Barnes and Pedersen, 1993[7]; Hanania et al., 1995[48]; Roland et al., 2004[107]). These additives are found in MDIs, and because DPI products do not contain these excipients, throat irritation and cough with DPI is less common (Barnes and Pedersen, 1993[7]; Roland et al., 2004[107]). To reduce the risk of this complication, spacers or bronchodilators such as beta-agonists can be used as a pre-treatment. Also, changing the MDI to DPI and reducing the respiration rate can be highly effective (Hanania et al., 1995[48]; Irwin and Richardson, 2006[53]). However, in a group study on the local effects of corticosteroids on children, it was found that cough was the most probable side effect (40 % of the treated population) and most often occurred with a spacer (Dubus et al., 2001[33]).

#### Perioral dermatitis and changes in tracheobronchial epithelium

Corticosteroids cause noticeable changes in the skin due to inhibition of fibroblasts activity and reduced collagen synthesis, but due to structural differences in the epithelium of the mouth and respiratory tract compared with the skin, these effects are negligible and insignificant in inhaled corticosteroids (Hanania et al., 1995[48]). The probability of this side effect depends on the type of device and its accessories; so that in use with spacer and a face mask about 5 % and in use with a nebulizer (regardless of the presence or absence of face mask) up to 14 % of the population might get involved (Dubus et al., 2001[33]; Roland et al., 2004[107]; Irwin and Richardson, 2006[53]). To treat this problem in severe cases, topical formulations of erythromycin or metronidazole can be used (Roland et al., 2004[107]).

#### Thirst

Thirst is one of the most obvious effects of inhaled corticosteroids, with a 20 % chance of occurring. Thirst can occur following throat irritation or candidiasis, but the most important risk factor is concomitant treatment with inhaled corticosteroids and long-acting beta-agonists (Dubus et al., 2001[33]; Roland et al., 2004[107]). Budesonide has been shown to be safer than beclomethasone in many local side effects, including thirst and cough (Irwin and Richardson, 2006[53]).

#### Tongue hypertrophy

Tongue hypertrophy is one of the rarest side effects of inhaled corticosteroids with a 0.1 % chance of occurring, most commonly associated with nebulizer use (Dubus et al., 2001[33]). The main cause of this complication, which is more common in children and infants, is the hypertrophy of the tongue muscle and the local accumulation of fat in this area (Roland et al., 2004[107]).

#### Respiratory infections

At normal doses of inhaled corticosteroids, there is no evidence of viral or bacterial infection or an increase in the number of pathogens present in the sputum. Higher doses require further evaluation and study (Hanania et al., 1995[48]).

### Systemic side effects of inhaled corticosteroids

Inhaled corticosteroids enter the systemic bloodstream after taking two different routes. Most of the drug is delivered to the lungs and enters the bloodstream based on the rate and extent of pulmonary absorption. Some portion of the drug remains in the mouth and throat and enters the gastrointestinal tract, in which case, after gastrointestinal absorption, the drug undergoes the first-pass effect and then the rest enters the circulation (Barnes and Pedersen, 1993[7]; Irwin and Richardson, 2006[53]). Systemic effects of inhaled corticosteroids often occur over a long period of time and depend on several different factors including dose, frequency of use, site of absorption, lipophilicity, individual differences, age, pharmacogenetics, and pharmacodynamics (Barnes and Pedersen, 1993[7]; Hanania et al., 1995[48]; Irwin and Richardson, 2006[53]). The shorter the drug is in the bloodstream and the sooner it is cleared from the blood, the less systemic side effects it will have. Budesonide, for example, has fewer side effects than beclomethasone due to its high clearance rate and rapid hepatic metabolism (Hanania et al., 1995[48]). Lipophilic corticosteroids such as fluticasone and mometasone have a low clearance rate due to high tissue uptake. Also, the higher the protein binding of the drug and its hepatic first-pass metabolism, the less it is likely to cause side effects (Irwin and Richardson, 2006[53]). Several recommendations can be made to reduce the systemic side effects of inhaled corticosteroids. Poor inhalation techniques cause the drug to remain in the mouth and throat, and as the drug enters the bloodstream from the gastrointestinal tract, the risk of systemic complications increases. To solve this problem, a spacer can be used to strengthen the breathing technique. Also, rinsing the mouth and throat area after each inhalation has a positive effect (Hanania et al., 1995[48]). As the delivery of the drug to the depths of the respiratory tract increases the efficacy of the drug and its systemic side effects increase. Therefore, the best solution is to reduce the dose of the drug so, it continues to have its maximum effect. For example, the use of formulations that use hydrofluoroalkane (HFA) instead of chlorofluorocarbon (CFCs) allows the drug to be placed better in the lungs and so, the dose can be reduced (Irwin and Richardson, 2006[53]). However, inhaled corticosteroids have fewer systemic side effects than systemic corticosteroids, and this is a great advantage (Dahl, 2006[23]). In the following, we will examine the systemic side effects of inhaled corticosteroids.

#### Hypothalamic-pituitary-adrenal (HPA) axis suppression

It has been shown that the use of oral and injectable corticosteroids can cause adrenal suppression by reducing the production of ACTH in the pituitary gland, and thus, reducing the secretion of cortisol in the adrenal gland. If this process continues, adrenal atrophy may be seen (Barnes and Pedersen, 1993[7]). Many studies have shown that this complication is also possible for inhaled corticosteroids and in fact, it is the most serious complication (Hanania et al., 1995[48]; Dahl, 2006[23]). This complication depends on the dose, duration of treatment, frequency of use, route of administration, and time of use (Barnes and Pedersen, 1993[7]; Dahl, 2006[23]). The lower the dose and frequency, the less adrenal suppression will occur. Also, the closer the consumption time is to 08:00 am, the less ACTH will be inhibited (Barnes and Pedersen, 1993[7]). Beclomethasone and budesonide suppress the HPA pathway at doses greater than 1,500 and 400 micrograms per day, respectively, but ciclesonide appears to have no effect on cortisol secretion (Hanania et al., 1995[48]; Dahl, 2006[23]). The degree of suppression of HPA axis by corticosteroids (determined by assessing changes in cortisol levels at different times of a day) is a good measure of the severity of other systemic complications (Dahl, 2006[23]).

#### Cataracts

There are disagreements about this complication in various studies. In general, posterior subcapsular cataracts are possible following steroid use. One report stated that there is a risk of posterior subcapsular cataracts (PSC) following the use of inhaled beclomethasone and dexamethasone, but this is considerably less likely than when these corticosteroids are taken systemically (Barnes and Pedersen, 1993[7]). Another study claims that taking inhaled corticosteroids has a very low risk of cataracts, even at high doses (Hanania et al., 1995[48]). In a review on the systemic effects of inhaled corticosteroids, it was stated that the risk of developing cataracts in children is minimal and increases with age, but there was no established link between them (Dahl, 2006[23]). Unlike others, another review article stated that taking inhaled corticosteroids can double the risk of developing PSC (Toogood, 1998[134]). It also links this risk to current and cumulative doses. More detailed studies are needed to determine the relationship between inhaled corticosteroid use and the incidence of PSC.

#### Glaucoma

Because intraocular pressure does not change much after inhaling corticosteroids, the risk of glaucoma is substantially low but should be monitored for long-term use (Dahl, 2006[23]). In a review article on the side effects of inhaled corticosteroids, it was explained that primary open-angle glaucoma can be exacerbated by inhaled corticosteroids, even at low doses, so the history of patients' family should be checked (Toogood, 1998[134]).

#### Effects on bone metabolism

Corticosteroids play an important role in bone formation by having a direct effect on osteoblasts and osteoclasts. Systemic corticosteroids have been shown to cause osteoporosis by increasing bone resorption and decreasing bone formation (Barnes and Pedersen, 1993[7]; Dahl, 2006[23]). Therefore, biochemical changes due to conversion in bone formation and resorption following the use of inhaled corticosteroids can be expected (Hanania et al., 1995[48]). However, research studies show that common doses in children are not associated with a decrease in bone density, and there is no significant reduction in bone mass density (BMD) in adults; but in long-term use, especially for people prone to osteoporosis such as postmenopausal women, this risk should be monitored regularly (Toogood, 1998[134]; Dahl, 2006[23]; Irwin and Richardson, 2006[53]). The risk of fracture for users of inhaled corticosteroids is minimal and there is no approved guideline to prevent such side effects. Beclomethasone has more adverse effects on bone than other corticosteroids and is not considered safe at this point (Toogood, 1998[134]).

#### Growth

Corticosteroids alter the growth process by inhibiting the synthesis of type 1 collagen, which is locally present in bones (Toogood, 1998[134]). In asthmatic children who use long-term inhaled corticosteroids, growth retardation is likely, especially in the lower leg. However, children's height growth is a multifactorial phenomenon, and this theory cannot be generalized (Dahl, 2006[23]). In fact, inhaled corticosteroids alter a child's growth pattern, but the final height appears unchanged so that the growth rate decreases compared with the placebo group, but the growth continues for a longer time (Barnes and Pedersen, 1993[7]). Growth abnormalities are less common in fluticasone than in budesonide and beclomethasone, and therefore, it appears safe for children in low to moderate doses (Toogood, 1998[134]). Also, ciclesonide up to 160 micrograms per day did not display any effects on inhibiting the growth of lower-leg in children for short-term use (Dahl, 2006[23]). Unlike inhaled prednisolone, budesonide has negligible adverse effects on children's growth (Barnes and Pedersen, 1993[7]).

#### Skin bruising

As mentioned earlier, corticosteroids reduce the activity of fibroblasts and inhibit the production of type 1 and type 3 collagen, which can eventually lead to thinning of the skin. This complication is possible following the long-term use of high-dose inhaled corticosteroids (Barnes and Pedersen, 1993[7]). In addition to the dose, duration of treatment, age, and sex of the patient also play a role in the occurrence of this complication. Elderly patients and women are more prone to it (Hanania et al., 1995[48]; Toogood, 1998[134]; Dahl, 2006[23]). Other cutaneous side effects of inhaled corticosteroids include steroid purpura, oral blood blisters, and acne, which are less common (Hanania et al., 1995[48]; Toogood, 1998[134]).

#### Metabolic changes

Unlike systemic glucocorticoids, usual doses of inhaled corticosteroids have no effect on fasting blood sugar or fasting insulin levels. Hemoglobin A_1c_ (HbA_1c_) is also remained unchanged. High-dose therapy can increase fasting insulin levels and peak glucose levels. High-dose treatment can increase fasting insulin levels and peak glucose levels. In contrast to beclomethasone, flunisolide seems to be a suitable choice in terms of not changing the fat profile (Barnes and Pedersen, 1993[7]).

#### Hematologic and immunologic changes

Inhaled corticosteroids exhibit various effects in the number of white blood cells (WBCs). For instance, in some studies they rose the number of neutrophils (Pasternak et al., 2016[96]), while in some other they had no effects on the neutrophils count with a decline in the number of eosinophils (Demarche et al., 2017[27]). At high doses of inhaled budesonide, a decrease in the number of monocytes was observed (Barnes and Pedersen, 1993[7]). Unlike systemic corticosteroids, inhaled corticosteroids do not significantly suppress immunity that would eventually lead to bacterial, viral, or fungal diseases, but the patient should be screened for tuberculosis (Toogood, 1998[134]).

#### Neuro-behavioral changes

Hyperactive behavior, aggression, insomnia, uncontrolled behavior, difficulty concentrating, depression, euphoria, nightmares, and drowsiness are possible in the first few days of inhaled corticosteroid usage (Barnes and Pedersen, 1993[7]; Hanania et al., 1995[48]; Taha, 2017[128]).

### Specific side effects of inhaled corticosteroids

#### Ciclesonide

Among corticosteroids, ciclesonide, in addition to its anti-inflammatory and immunosuppressive effects, also has anti-viral properties. Ciclesonide and mometasone can disrupt viral replication as much as lopinavir. Ciclesonide inhibits the viral replication of human coronaviruses such as HCoV-229E and SARS-CoV and other positive-strand RNA viruses such as rubella virus (Halpin et al., 2020[47]; Matsuyama et al., 2020[77]; Saghazadeh and Rezaei, 2020[111]). For this reason, treatment with this drug can be significantly more effective and different from other corticosteroids. According another computational hypothesis, the anti-coronavirus effect of ciclesonide could be due to its interference with the active sites in the SARS-CoV-2 NSP15 endonuclease (Kimura et al., 2020[62]). In patients with COVID-19 with symptoms of non-severe pneumonia who took ciclesonide experimentally, the number of lymphocytes increased significantly and it was demonstrated that ciclesonide could inhibit the exacerbation of COVID-19 (Yamasaki et al., 2020[143]). In another study, three patients experienced successful treatment with inhaled ciclesonide due to the combined antiviral and anti-inflammatory effects of this compound (Iwabuchi et al., 2020[54]). Also, triple combination therapy with ciclesonide, azithromycin and hydroxychloroquine for 5 patients, started on the seventh day of the onset of symptoms, improved the patients' condition in less than 5 days (Mori et al., 2020[83]). Extensive research has been conducted on the use and side effects of ciclesonide, and it has been shown that the drug is well-tolerated in adults and adolescents. No significant changes were found in cortisol and creatinine levels in ciclesonide therapy (Larsen et al., 2003[63]). Side effects are mild to moderate and often include nasopharyngeal events, including oral candidiasis and hoarseness of voice. Other possible side effects include asthma, rhinitis, headache, and upper respiratory tract infections (Reynolds and Scott, 2004[102]; Dahl, 2006[22]; Deeks and Perry, 2008[26]).

#### Triamcinolone

The use of nebulized triamcinolone after intravenous dexamethasone administration in COVID-19 causes the anti-inflammatory effect to be concentrated in the lungs (Theoharides and Conti, 2020[130]). Triamcinolone is available in various dosage forms in the pharmaceutical market and is commonly used for its topical anti-inflammatory effects, but for its inhaled form, a mild systemic effect can be considered (Fabbri and Melara, 2001[36]; Falk et al., 2008[37]). According to a report, the use of triamcinolone in the form of oral paste is somewhat effective in correcting the olfactory (anosmia) and taste dysfunction in COVID-19 patients (Singh et al., 2021[123]). The risk of adverse gastrointestinal events of triamcinolone is often lower than other corticosteroids and is a good choice for patients with a history of gastrointestinal problems. However, severe osteoporosis has been reported in some cases of triamcinolone therapy. Headache and epistaxis are also major side effects of triamcinolone acetonide. Other side effects such as throat discomfort, dizziness, light-headedness, sleepiness, flushing of the face and trunk, lack of appetite, nasal irritation, sneezing, nasosinusal discomfort, and dryness of the mucous membranes are also possible. Research studies showed that taking triamcinolone, contrary to other corticosteroids, can help losing weight (Golding, 1960[45]; Jeal and Faulds, 1997[58]). There is a possibility of a variety of infections, including fungal infections, but the incidence is very low, and it is a minor complication. Each of these side effects is more likely to occur in the inhaled dosage form than in oral consumption (Jeal and Faulds, 1997[58]).

#### Beclomethasone

Beclomethasone dipropionate at a dose of 400-800 micrograms per day is a common treatment for asthma patients. Histological examination shows that mucosal damage is minimal with inhaled beclomethasone. Oropharyngeal candidiasis, hoarseness, PSC, respiratory infections, cough, thirst, osteoporosis, skin bruising, wheezing, dysphonia, local irritation, and sore throat are common side effects of beclomethasone dipropionate (Brogden et al., 1984[13]; Shim and Williams, 1987[120]; Shaikh, 1992[118]; Toogood, 1998[134]; Lipworth, 1999[71]; Dubus et al., 2001[33]; Roland et al., 2004[107]; Irwin and Richardson, 2006[53]). Long-term use of doses above 1000 micrograms per day increases the likelihood of adrenal suppression (Brogden et al., 1984[13]).

#### Budesonide

Budesonide is a non-halogenated glucocorticoid that can be inhaled at a dose of 200-800 micrograms per day. Nebulized budesonide has been shown to improve oxygenation and reduce inflammatory markers such as TNF-α, IL-1β, and IL-6 in patients with acute respiratory distress syndrome (Miyazawa and Kaneko, 2021[81]). In order to control the symptoms of asthmatic patients, inhaled budesonide can be gradually replaced by oral prednisolone, because it has less adrenal suppression and is sufficiently effective (Clissold and Heel, 1984[19]). At regular doses, budesonide is generally well-tolerated, but side effects such as oral candidiasis, dysphonia, skin bruising, osteoporosis, slowing lower-leg growth, subcapsular and nuclear cataract, adrenal suppression, and sore throat have been reported (Clissold and Heel, 1984[19]; Toogood, 1998[134]; Lipworth 1999[71]; Dahl, 2006[23]; Ding et al., 2016[32]). Increased alkaline phosphatase, decreased leukocyte count, and decreased serum bilirubin levels are also important laboratory findings following the use of inhaled budesonide. Inhalation of budesonide up to a dose of 800 micrograms does not alter cortisol levels, but at higher doses, despite high therapeutic efficacy, adrenal suppression can be expected. Complications such as hoarseness, sore throat, exacerbation of eczema, steroid related psychosis, pulmonary eosinophilia and sarcoidosis, arthralgia, and myalgia are possible following replacement of oral corticosteroids with inhaled budesonide (Clissold and Heel, 1984[19]).

#### Fluticasone

Fluticasone may inhibit the integration process by affecting ACE2, the main entrance receptor for the SARS-CoV-2 (Cava et al., 2020[17]). Fluticasone has also been used to treat anosmia caused by COVID-19 (Singh et al., 2021[123]). Among inhaled corticosteroids, fluticasone propionate has the highest lipophilicity, potency, half-life, primary hepatic metabolism as well as the highest systemic bioactivity, and therefore, has the highest adrenal suppression rate. Due to the long half-life of fluticasone, repeated administration instead of once a day will double adrenal suppression (Lipworth, 1999[71]). Side effects reported with fluticasone propionate include adrenal suppression, increased intraocular pressure, glaucoma, posterior subcapsular cataracts, skin bruising and osteoporosis, which often occur at high doses (Toogood, 1998[134]; Lipworth, 1999[71]).

#### Mometasone

In addition to its appropriate anti-inflammatory effect, mometasone has a direct anti-viral replication effect with a different mechanism of action than ciclesonide. Another advantage of mometasone is the smaller particle size than budesonide, which improves efficacy and reduces local side effects (Miyazawa and Kaneko, 2021[81]). Mometasone furoate at a dose of 100-400 micrograms twice daily is one of the most common treatments for respiratory patients. Common side effects of mometasone DPI and MDI forms include dry mouth, mouth ulceration, pharyngitis, upper respiratory tract infections, cough, hoarseness, dry throat, rash, dyspepsia, oropharyngeal pain, headache, dysphonia, and oral candidiasis (Bernstein et al., 1999[9]; Price et al., 2010[98]; Nolte et al., 2013[92]).

#### Flunisolide

Flunisolide, which is often used to treat allergic rhinitis, activates the glucocorticoid receptor and has good potency in inhibiting inflammation by inhibiting alveolar macrophages (Nunnari et al., 2020[93]). This cytochrome P450 inhibitor appears to be safer than other inhaled corticosteroids because almost no side effects were found in a mass study designed to determine its effect (Newman et al., 1998[88]). In another study, transient side effects such as headache, anxiety, and palpitations have been observed following the use of flunisolide (Rodrigo and Rodrigo, 1998[105]). Nonetheless, more comprehensive and extensive research is needed to elucidate the details of the adverse events of this drug.

## Conclusion

Several parameters are considered to compare the performance of corticosteroids with their side effects in the treatment of COVID-19. The duration of action of corticosteroids varies and is divided into three categories of long-acting, intermediate-acting, and short-acting. Betamethasone and dexamethasone have the longest duration, and hydrocortisone and cortisone have the shortest duration of action among corticosteroids. The potency of each drug is also important in choosing the type of treatment and its effectiveness. Dexamethasone and betamethasone are the strongest, and cortisone and hydrocortisone are the weakest drugs in terms of potency. For this reason, a series of low-potency drugs like cortisone and hydrocortisone in low doses may not be enough to curb the cytokine storm and the immunological incidences caused by the body's exposure to the virus. The protein binding of each drug is also effective in drug interactions and beneficial performance. Most corticosteroids have a high percentage of protein binding. In patients with no history of cardiovascular problems, fludrocortisone is a good option because its side effects are more limited than glucocorticoids. Despite its high potency, dexamethasone is often tolerable but should be used with caution in gastrointestinal cases. Recent research has shown that dexamethasone is a very effective drug to prevent fatalities in critically-ill patients (Jamaati et al., 2020[56]). Hydrocortisone has very mild side effects and is well tolerated by patients, but more research is needed to prove its effectiveness in dealing with problems such as cytokine storms. Inhaled corticosteroids due to their high adequacy along with very limited side effects can be new and suitable options to prevent mortality in coronavirus pandemic. Ciclesonide is a suitable drug due to its limited side effects and sufficient potency, but its common dosage form is inhaled, which may limit its use during shock and cytokine storm. The other corticosteroids listed may be used in specific situations, and it is important to consider the patient's clinical profile and other health conditions and adapt the therapeutic regimen to the common side effects of each of these medications.

## Acknowledgement

We would like to thank the department of Medicinal Chemistry and Toxicology and Pharmacology, Faculty of Pharmacy, Kerman University of Medical Sciences for their sincere help in writing this article.

## Funding

This research did not receive any specific grant from funding agencies in the public, commercial, or not-for-profit sectors.

## Conflicts of interest

The authors declare that they have no competing interests.

## Author contribution

Mohammad Amin Langarizadeh: Writing, reviewing and conceptualization; Marziye Ranjbar Tavakoli & Ali Ghasempour: Data curation and original draft preparation; Ardavan Abiri: Editing and validation; Masoud Rezaei: Visualization and designing; Alieh Ameri: Methodology and supervision.

## Figures and Tables

**Table 1:**
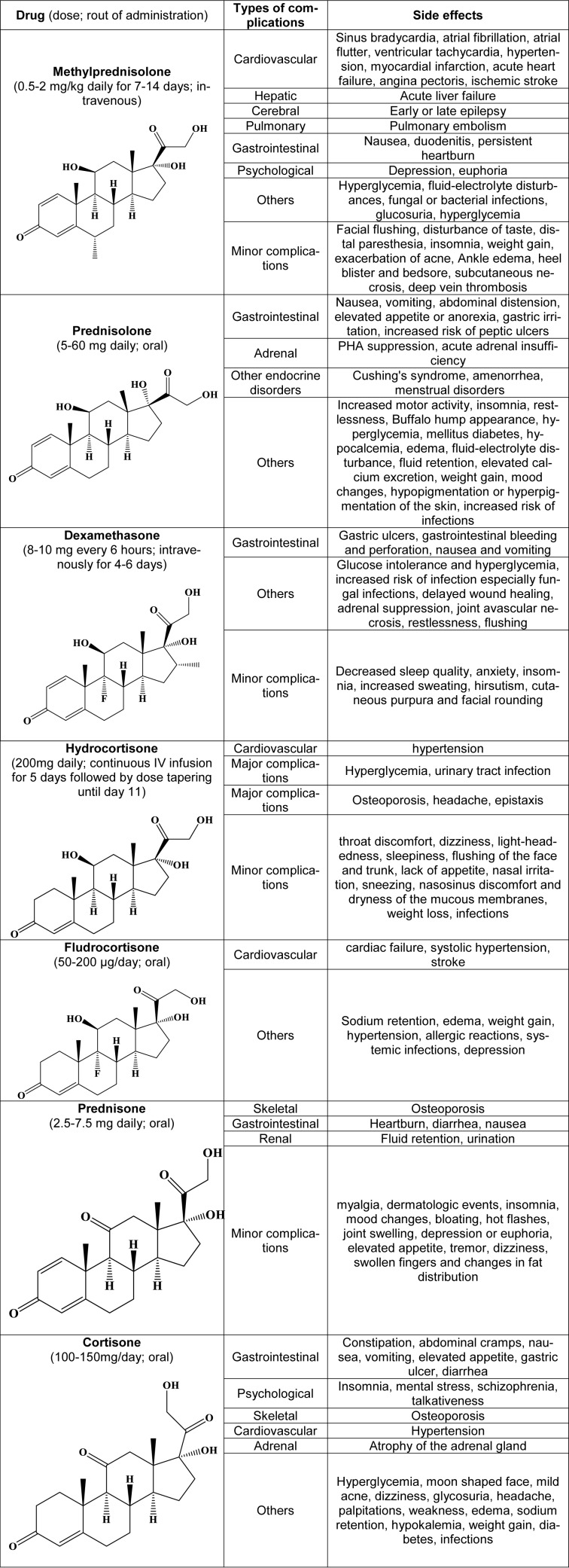
Corticosteroids used in COVID-19 with their specific side effects
